# Causes and Modes of Communication of Contagious and Infectious Diseases1Read before the Buffalo Academy of Medicine, Tuesday evening, September 12, 1893.

**Published:** 1893-11

**Authors:** Edward Clark

**Affiliations:** Buffalo, N. Y., Ex-Health Physician


### CAUSES AND MODES OF COMMUNICATION OF CONTAGIOUS AND INFECTIOUS DISEASES.[1]

By EDWARD CLARK, M. D., Buffalo, N. Y.,
Ex-Health Physician.

THE Causes and Modes of Communication of Contagious and Infectious Diseases, the subject allotted me this evening, is one of such vast importance, and so deserving of our best thought and attention, that it will be impossible for me to do any sort of justice to it in a ten-minute paper. This, together with the fact that I am acting as a substitute tonight for our able Health Commissioner, and have had only four or five days in which to prepare my remarks, must be my apology to you for this, perhaps, somewhat rambling and incomplete discourse. The words contagious and infectious, as applied to disease, have given rise to no little confusion among physicians, as to their proper scope and meaning. That they are synonymous terms is held by many, while others argue that each has a distinct and separate meaning.

Personally, I regard contagious as a more comprehensive term than infectious, and believe it may even include the latter. The difference in meaning, however, of the two words, has no practical significance, for the efforts of physicians and sanitarians are called frequently into requisition to check and conquer both forms of disease. To indicate a typically contagious disease and a typically infectious disease, I might mention small-pox, contagious, and typhoid fever, infectious.

The chief and distinguishing characteristic of a contagious or infectious disease is its specificity; that is, the disease transmitted

---

1. Read before the Buffalo Academy of Medicine, Tuesday evening, September 12, 1893.

or caused by the particular virus of that disease is always the same in its essential characteristics. It may be of a very mild type, or of a very severe one, but, so far as quality is concerned, is always the same disease from which it sprung.

"Men do not gather grapes from thorns nor figs from thistles," and small-pox always produces small-pox, scarlet fever always produces scarlet fever, and typhoid fever always produces typhoid fever. No matter how light the attack may be of any of these diseases, the virus or contagion produced is such that it will give rise to the same disease in another, and here the attack may be either very mild or very severe, its character depending, no doubt, on some inherent condition of the receptive party. A case of small-pox, scarlet fever, or diphtheria, which is so mild that it does not confine the patient to bed, may give rise to a form of the same disease in others of the most malignant type. In fact, I regard these "walking cases," so-called, as the most dangerous, so far as the spread of the disease is concerned, because, as a rule, the restrictions and confinement imposed upon the patient are less complete, and the precautionary measures less secure, than in cases where the type of disease is more severe. From a large personal experience with small-pox, I know that the mildest possible cases should be subjected to a rigid isolation and complete quarantine until all danger is past, and I think the same rule should apply to other contagious diseases, especially scarlet fever and diphtheria.

The fact that all contagious and infectious diseases are the progeny of their own kind, and do not originate *de novo*, is, to my mind, the most unanswerable and convincing argument in favor of their germal or bacillary origin. I think I can affirm, without fear of successful contradiction, that by far a large majority of the best observers and experimenters, both at home and abroad, believe, as I do, that each and every form of contagious and infectious disease is produced by a specific germ peculiar to that disease, and to no other. "Hence, the one great fundamental idea, which it is important for the laity, as well as the members of our profession, to grasp, is that the "germ theory" of disease is now established on a firm scientific foundation," and this supplies us a basis on which to erect the grand superstructure of preventive medicine. If this is so, it follows that all the diseases of the above-named types are preventable, and we will be effective with our preventive measures when we are fully able to recognize and study the life history of their causative germs.

Among the diseases which physicians have to deal with, under the head of contagious and infectious, we may mention: scarlet fever, diphtheria, small-pox, measles, pertussis or whooping cough, parotiditis, typhoid fever, cholera, yellow fever, typhus fever, glanders, epidemic cerebro-spinal meningitis, and tuberculosis, both general and local. As many of the above are somewhat rare with us, and as my time is limited, I shall take up, for consideration tonight, only five of the above-named diseases, four of which—scarlet fever, diphtheria, typhoid fever, and tuberculosis—are quite prevalent in our climate, and one, cholera, which is attracting much attention at the present time, and which we hope will not reach our city. Let us first consider diphtheria. Perhaps no disease in modern times has attracted more attention than this, and the clinical and experimental studies of Brettoneau, Trosseau, Virchow, Oertel, Mackenzie, Klebs, Wood, Formad, Sternberg, Loeffler, Prudden, Northrup, Koch, and many others of recent times, in and by strictly scientific methods, have elucidated the fact that true diphtheria is caused by a germ now known and described as the Klebs-Loeffler bacillus.

The type of disease produced by this particular organism may be either severe or mild, and in either case is liable to be followed by constitutional symptoms, such as the various forms of paralysis, etc. There is a type of pseudo-membranous disease, affecting the throat and air passages, which is caused by the streptococcus and other cocci. This disease has been called true diphtheria by many, but it is not, and it is not followed by the sequelæ which we often see in cases of disease caused by the Klebs-Loeffler bacillus. This bacillus, the true cause of diphtheria, not only possesses remarkable vitality, but remarkable powers of propagation. Numerous instances are on record where objects infected months and years previously, have communicated diphtheria. While filth does not of itself cause diphtheria or any other infectious or contagious disease, it affords the best possible soil for the development and growth of their deadly germs. Currents of air, or gases ascending from cess-pools, foul vaults and sewers, carry the germs and propagate diphtheria. In any large city like ours, where we have diphtheria, the many miles of underground sewers afford a good place for the propagation of the germ, and sewer gas, carrying the germ, and escaping into apartments, and inhaled by children, has caused diphtheria in numerous instances. This is why health officials are so particular about having good sewage and good plumbing, for the records of the health departments of any large city teem with instances where death and sickness have entered many homes through the avenues of untrapped pipes and improperly ventilated sewers. In fact, the rules for the construction of this class of work cannot be too severe or too rigidly enforced. Whenever diphtheria is endemic or epidemic, we find a certain number of mild cases—walking cases, so-called, which spread the disease very largely; they are not sick enough to be confined, and are allowed to go about among other children, sometimes even to school. The cases arising from these mild cases may be of the most malignant type, the severity, no doubt, depending on the particular condition of the individual affected. The following instance, showing a common mode by which diphtheria originates in cities, is related by Mallin: A boy, ten years old, had diphtheritic angina, followed by almost universal paralysis; the room which he occupied was found to contain an offensive odor, which was traced to an untrapped and broken pipe communicating with a cesspool. There was no diphtheria in the neighborhood, and no other cases occurred in the family. It seemed certain that the bacillus came from the drain. Domestic animals and fowls are subject to the disease in question, and they undoubtedly convey the infection to human beings to a greater or less extent. Dogs, cats, cows, calves, swine, goats, rats, mice, and especially the feathered tribes, are said to be subject to the disease, and they not only convey the disease by direct contact, but are the carriers of the contagion in their hair and feathers. Of these, the dog and cat exert the greatest influence for evil, as they are quite likely to be fondled by children suffering from the disease.

Air, water, and food, and especially milk, are the media through which the virus enters the body, and the mouth, throat, and air-passages are the localities where the germ lodges and develops, although inoculation may occur through any wound or abrasion on the body. It is not necessary that the germ be swallowed to produce the disease, as is the case with the germs of cholera and typhoid fever. A great variety of conditions increase the susceptibility of some persons, such as nasal and pharyngeal catarrh, debility from any cause, age, etc. Some assert that scrofulous children with light hair and eyes, contract the disease quicker and suffer from its ravages greater than those having dark hair, eyes, and skin. The disease is not caused by any agency within the body, but always comes from without.

The paralysis and other sequelæ are said not to be produced by the bacillus invading the blood channels, but by the noxious substances or ptomaines evolved by the bacillus. The ptomaines of diphtheria are among the most deadly poisons. One great reason why diphtheria prevails most during the cold, damp months of Autumn and early Spring, is, perhaps, that catarrhs and other throat troubles are more prevalent during these months, and the germs of diphtheria find easier access to the system on account of the already diseased conditions of the mucous membranes. In the Annals of the Pasteur Institute, "Roux and Yersin state it as their opinion that the Klebs-Loeffler bacillus is so specifically identified as a cause of diphtheria, as to render it necessary that all practicing physicians should be able to isolate and identify this bacillus. They think that not until diagnoses are made in this way will thoroughly scientific results be attained." "In order to stain the bacillus so that it can be readily seen and studied under the microscope, it is merely necessary to remove a small fragment of the false membrane, by means of a piece of absorbent cotton wool, tied firmly to a pair of forceps, or any other safe carrier, from which it is transferred to a scrap of blotting paper, and thence to a cover glass, when it is broken down as finely as possible, heated over a flame and stained methyl olive or gentian violet, washing thoroughly with water before examining."

Of the exact nature of the specific cause of scarlet fever, we have not so thorough a knowledge as of that of diphtheria, but observation and research have given us much insight into its behavior. The evidence is very strong that the disease in question does not originate *de novo*, or that it does not spring from certain atmospheric or telluric conditions, but is produced by a definite specific principle, since countries have been free from it for centuries, till it was imported by commerce. That it sometimes appears in certain localities and persons without any known exposure, can be explained by the fact that the causative bacillus is so subtle and transmissible that it is conveyed long distances in articles of merchandise, even in very small packages. Reading-matter and letters going through the mails can carry it ; the dry goods in the stores, the fruits in the groceries, and the milk from the dairies, are looked upon as carriers of the contagious principle. The newspapers delivered at our doors, and the hand-bills thrown upon our doorsteps, may serve as vehicles for conveying the disease. The furniture, carpets, and even the wall paper of a room used by a scarlet fever patient, have been known to hold and transmit the disease even after a long period of time has elapsed. Domestic animals, especially the dog and cat, may carry the germ in their fur. Doctors in attendance on scarlet fever patients, unless extremely careful, can carry the microbe in their hair, and beard, and on their clothing. Currents of air and flies can also carry the contagion; in fact, the only way, perhaps, in which it cannot be conveyed, is by telegraph or telephone. The specific cause of scarlet fever is probably reproduced in the throats of patients, and in the desquamation from the skin. The sputa and other secretions from the mouth and throat dry, and thus the germs are set free in the air. The dried secretions, then, are just as dangerous in spreading the disease as are the small scales or particles thrown off in the process of desquamation. The disease is undoubtedly caused by the inhalation and lodgement of these particular specific particles in the throat of the victim.

As compared with other diseases, the one which must be recognized on every hand as being par excellence, that to which sanitarians and physicians must devote their special attention, is tuberculosis. This disease, or consumption, is produced by a bacillus which behaves to a great extent very much as does that of scarlet fever and diphtheria; this is especially true of that form which affects the lungs. The germs are coughed up with the sputa; these dry and set the bacillus free in the atmosphere.

This is the greatest and most dangerous source from which the disease springs. When we see a number of consumptive cases following each other in the same family, and where we see the husband or wife of a consumptive marital partner developing the disease, we may be quite sure that the disease came on through inhalation of the specific germ. Swallowing the germ may sometimes produce tubercular trouble in the bowels and rectum, and it is said to occur locally through a process of inoculation, but by far the large majority of cases are those of the pulmonary type, and are produced by drawing the bacillus into the lungs with the inspired air. It is said that the germ is not so tenacious of life as the scarlet fever bacillus, so that it cannot be so readily transported, but that it is carried to a limited extent by the same agencies, and in same manner is quite probable. Where the disease affects the intestines and rectum, the discharges from the bowels may contain the bacillus, and these, in consequence of improper drainage and sewer facilities, may contaminate water supplies. Local lesions of a tubercular character may also act as foci of infection.

Many of the lower animals suffer from tubercular disease, especially the bovine species, and it is quite well established, or at least believed by many, that the disease from them can be transmitted to the human family in their milk and meat. This, however, is such an exhaustive subject, that I can give it only a passing allusion. Its importance, however, cannot be overestimated, and it is being made a special study by many noted sanitarians of to-day, as it has in times past. At the present day, almost all sanitary authorities agree that the beef from tuberculous cattle and the milk from tuberculous cows should be prohibited as unfit for food. The milk which gives rise to tuberculosis is probably that from cows suffering from tubercular disease of the udders, known as tubercular mammitis. While exact statistics are not at hand, it is asserted upon pretty good authority that over forty per cent. of dairy cattle in this country are afflicted with tubercular disease, and the finer grades of cattle are said to be the greatest sufferers. If this is so, we have here a very powerful etiological factor, and it behooves us as sanitarians and physicians to give this whole subject very serious attention. This subject is full of interest. But time is hastening, and for fear that I am wearying you I will hasten to briefly allude to typhoid fever and cholera before closing this paper; but before leaving this subject I wish to mention one fact, which shows a decided difference in the character of such infectious diseases as scarlet fever and diphtheria, and tuberculosis on the one hand, where the bacillus is inhaled, and such diseases as typhoid fever and cholera on the other hand, where the bacillus is swallowed and gains entrance into the intestinal canal. This fact or proposition is that, as a rule, all inflammatory diseases of the throat, air-passages, and lungs, are increased by exposure to a cold atmosphere; that the same is true of all specific diseases which generally enter the body by way of the throat, air-passages, or lungs. On the contrary, diseases which generally enter the body by way of the mouth and alimentary canal, increase during or following exposure in a warm atmosphere.

"It is now pretty conclusively established that the specific cause of typhoid fever is reproduced in the intestinal canal, and sometimes in other parts of the body, having been found in the spleen and other organs." The characteristic lesion found in the lower part of the small intestine, indicates in what part of the body the germ is most destructive of tissue. The glandular apparatus of the small intestine is undoubtedly concerned somewhat in this ulcerative process, for the destruction of tissue takes place only over an aggregation of the solitary glands of the bowel, known as Peyer's patches. The numerous experiments which have been made, with reference to a study of the bacilli of enteric fever, seem to point out the fact that both the bacillus coli communis, and the bacillus of Eberth, are instrumental in producing the disease.

"Arloing has reported the results of a study of the bacillus coli communis, and the bacillus of Eberth, made by Rodet, Roux, and Vallet. In an examination of the water supply of a community, in which 119 of the 215 persons were attacked with enteric fever, Rodet found an organism that possessed almost all of the characteristics of the bacillus of Eberth. Examination of two other water supplies used by communities in which enteric fever was prevalent, disclosed the presence of organisms that resembled the colon bacillus more closely than the bacillus of Eberth. Roux made a corresponding observation. Rodet and Roux now studied the stools of a number of enteric fever patients, and found some cases in which only the colon bacillus was present in the stools, and other cases in which the bacillus of Eberth was found in the spleen and the colon bacillus in the intestines. It was not possible to make a distinction between the two organisms from a study of their morphology, biology, or pathogenicity. These and other experimenters seem to have arrived at the conclusion that the colon bacillus and the bacillus of Eberth represent two species of the same organism, and that the human economy favors the transformation of the colon variety into the Eberth variety."[1]

Drinking water, contaminated by fecal discharges from typhoid fever patients, is, perhaps, the most common source or vehicle of typhoid fever. The disease has been traced to milk diluted with infected water, and apparently, in some cases, to emanations from cesspools and sewers. That the disease is sometimes caused by the inhalation of the specific microörganism seems to have been proven. At the present time, however, the weight of opinion seems to be that the specific germ must be swallowed, and thus

1. Caton, of Liverpool, calls attention to a possible means of dissemination of enteric fever from the employment of liquid manure obtained from cesspools, in the cultivation of lettuce, celery, and other vegetables.

gain an entrance into the stomach and small intestine. And the way in which this usually comes about, is that the germ is swallowed with our food and drink. The literature of the disease teems with illustrations of this fact.

The work of Koch and other investigators has left little doubt in the minds of the profession as to the true cause of cholera and its method of action. Like typhoid fever, the germ or bacillus is conveyed very largely by food and drink supply. Like it, also, it must find its way into the gastro-intestinal canal. Therefore, it must be swallowed, and not inhaled, to produce its specific effect. It is endemic only in some parts of Asia, and when other countries have it, it is always carried along the lines of commercial intercourse by land or water. Its diffusion has no relation to the direction or velocity of the wind. On land it creeps from place to place, and never invades an inland town or seaport without having been carried by some person or agency from a place already affected with the disease. A high atmospheric temperature is everywhere associated with the prevalence of cholera; its origin in a hot climate proves this beyond a doubt. The disease disappears to a great extent in very cold weather, and attains its greatest intensity in the hot summer months. Difference in temperature explains the fact, that of two ships arriving from Havre, France, in December, 1848, one at New York and the other at New Orleans, the former did not disseminate the disease, but the latter formed the starting point of an epidemic which lasted all Winter.

Much has been said and written about the predisposing causes of cholera, such as poverty, over-crowding, filth, intemperance, depression of spirits, etc.; that they all have a certain influence, cannot be doubted. They are the same factors which favor the spread of all infectious and contagious diseases. They never produce them *per se*, but simply afford powerful aids to the growth and development of their germs. This is especially true of overcrowding and its usual accompaniment—filth.

Some of the infectious diseases thrive better on filth than others. This is notably true of cholera and typhoid fever. One of the most graphic exhibits of the relations of filth to cholera and to typhoid fever is that prepared by Dr. Joseph von Foder, of Buda-Pesth, who tabulated the results of his investigations and study of the subject in Buda-Pesth for a period of fifteen years, 1863 to 1877 inclusive, as follows:

TYPHOID FEVER AND CHOLERA IN BUDA-PESTH, 1863 TO 1877.

1. *Influence of filthy houses:*

| | | |
|---|---|---|
| Deaths from cholera per 100 houses when the interior of the dwelling was..... | 1. Very clean..... | 92 |
| | 2. Clean ......... | 199 |
| | 3. Dirty ......... | 268 |
| | 4. Very dirty..... | 402 |
| Deaths from typhoid fever per 100 houses when the interior of the dwelling was. | 1. Very clean..... | 165 |
| | 2. Clean ......... | 177 |
| | 3. Dirty ......... | 182 |
| | 4. Very dirty..... | 356 |

2. *Influence of filthy yards:*

| | | |
|---|---|---|
| Cholera deaths per 100 houses when the yard was........................ | 1. Very clean..... | 188 |
| | 2. Clean ......... | 214 |
| | 3. Dirty.......... | 263 |
| | 4. Very dirty..... | 389 |
| Typhoid fever deaths per 100 houses when the yard was................. | 1. Very clean..... | 159 |
| | 2. Clean ......... | 186 |
| | 3. Dirty.......... | 208 |
| | 4. Very dirty..... | 282 |

The specific cause of cholera, then, is taken into the alimentary canal, and through it produces the specific and characteristic symptoms of the disease. It is conveyed from the sick to the well by the discharges from the bowels and the vomited matter of the persons affected; that is, the food and water supply becomes contaminated with these discharges, and are thus swallowed by the patient. The virus, or germ, can be carried in clothing and articles of merchandise, and is said to preserve its vitality for a long time. Personal wearing apparel of persons who are not cleanly in their habits affords a good vehicle for the transportation of the specific poison. There are good reasons for believing that the poison does not enter the system through the lungs. If the bacillus floats in the air, and is thus taken into the mouth, it must needs be swallowed to produce its specific effect.

I must leave the subject here, not because it is uninteresting, but because it is too comprehensive for one evening's discussion.

It is a subject on which volumes have been written, and at the present time is receiving more attention from sanitarians and law makers than ever before in the history of the human race. In fact, all departments of sanitary science are being studied by physicians, legislators, and the people, with an eagerness that is truly astonishing. The people, with the physicians, are coming to believe that preventive medicine is to be the medicine of the future, and that the chief end and aim of many doctors of the future will be to restrict and limit the progress of disease by the application of the principles of preventive medicine. It is a noteworthy truth, that "an ounce of prevention is worth a pound of cure," and the people who pay the taxes are awaking to the fact that it is much cheaper to keep disease out of a given locality than it is to fight it after having once gained an entrance. The losses and hardships entailed by the present financial distress in our country, are a mere trifle compared to those which would result if cholera should become epidemic within our border. The intelligent portion of our people are always, as a rule, ready to aid and coöperate with our health authorities in preventing and stamping out disease when the necessity and wisdom of such coöperation is clearly shown to them.

They realize that private or personal hygiene is good so far as it goes, but that it is *per se* totally inadequate to restrict and prevent any of those diseases which now cause the largest death-rate. "For protection against the dangerous communicable diseases, the general coöperation of all classes of people is essential. Each person is so continuously exposed, directly or indirectly, to other persons in all ranks of life, that no person can live to himself alone. The safety of each, in consequence of the many and insidious means by which contagious and infectious diseases are transported and communicated, is bound up with the safety of others, so that each individual has a vital interest in the general health, and in general and special measures for the preservation of the public health." In view of these facts, it is evident that the public health, and all matters pertaining thereto, must be looked after by the public health officials, aided and backed up by a vigorous and intelligent public press and public opinion. Lord Beaconsfield gave utterance to a great truth when he said: "The health of the people is the first duty of the statesman."

This forms a pleasing contrast to the opinions held by the people not many years ago, "when the most enlightened public view of the subject of general sanitation included not much more than the abatement of nuisances and the restriction of small-pox." "We know, however," and intelligent people to a large extent share our knowledge, "that the important diseases which are spread by ordinary filth are few compared with those which are spread by particular 'specific' causes, which cannot be recognized by any of the senses unaided." The specific causes of some of the most dangerous diseases have no odor. They are not visible, except through the aid of the microscope, or of some special method of cultivation, requiring the services of a skilled bacteriologist. Sanitarians have learned the cause of "the pestilence which walketh in darkness and wasteth at noonday," and are able to set forth to the people methods which will, undoubtedly, be effective for the prevention of that dread scourge, tuberculosis, or consumption, that causes more deaths than any other and of several other pestilences, which, like diphtheria and scarlet fever, rank only a little below that great white plague.

Is it too much to hope and believe that the rapidly revolving wheels of time will bring the human race, by-and-by, to a period when, as a result of patient study and laborious research, they will be able, fully and absolutely, to recognize the cause of each and every type of contagious and infectious disease? If the cause be a germ, or bacillus, as we are all coming to believe, will they not be able to study its method of development and propagation? This much accomplished, will it not be a comparatively easy task to compass the absolute destruction of these deadly germs, and forever wipe from the face of the earth the terrible scourges which now afflict the human family in the shape of what we now know as the contagious and infectious diseases?

